# Small dense low-density lipoprotein cholesterol was associated with future cardiovascular events in chronic kidney disease patients

**DOI:** 10.1186/s12882-016-0358-8

**Published:** 2016-10-06

**Authors:** Hao Shen, Yan Xu, Jingfen Lu, Chunfang Ma, Yadong Zhou, Qiong Li, Xu Chen, Ao Zhu, Guorong Shen

**Affiliations:** 1 Department of Clinical Laboratory Medicine, First People’s Hospital of Wujiang District, Nantong University, Suzhou, China; 2 Department of Nephrology, First People’s Hospital of Wujiang District, Nantong University, Suzhou, China; 3 Department of Geriatrics, First People’s Hospital of Wujiang District, Nantong University, Suzhou, China

**Keywords:** Chronic kidney disease, Cardiovascular diseases, Lipids, sdLDL-C

## Abstract

**Background:**

Cardiovascular disease (CVD) is often comorbid with chronic kidney disease (CKD). Small low-density lipoprotein cholesterol (sdLDL-C) has also been found to increase risk for CVD. The goal of the present study was to determine the nature of the relationship between sdLDL-C concentration and CVD in patients with CKD.

**Methods:**

One-hundred and forty-five subjects (113 men and 32 women) with CKD (Stage 3 and Stage 4) participated this retrospective study. The concentration of sdLDL-C was measured in samples from 145 CKD patients between 2010 and 2012 during a four-year follow-up period.

**Results:**

A total of eight fatal cardiovascular events (CVs) and 46 nonfatal CVs were registered in the four-year follow-up period. Multivariate Cox regression analysis showed that sdLDL-C [1.278, 95 % (1.019–1.598)] and sdLDL-C/LDL-C [2.456, 95 % (1.421–15.784)], at final observation, were independent risks of CVs. A Kaplan-Meier survival analysis showed that patients with sdLDL-C >38 mg/dl (logrank: 4.375, *P* = 0.037), and sdLDL-C/LDL-C ratio >0.3 levels (logrank: 11.94, *P* = 0.018) were at increased risk for CVs.

**Conclusion:**

The results of this study indicated that for patients suffering CKD, a significant relationship exists between an elevated sdLDL-C concentration and the risk of cardiovascular disease.

## Background

Chronic kidney disease (CKD) progression is frequently complicated with dyslipidemia, which is recognized as the most important risk factor causing cardiovascular disease (CVD) in CKD patients. Multiple observational studies have shown that low density lipoprotein cholesterol (LDL-C) is an effective, independent predictor of CVD morbidity and mortality; Small dense LDL (sdLDL) has been associated with increased risk for CVD in a number of cross-sectional studies [1–4]. This is most likely because in large quantities, sdLDL particles are more atherogenic than larger, buoyant LDL-C particles [2, 5].

Though the pathogenesis for the elevated risk of CVD in patients with CKD remains elusive, CVD is indeed the leading cause of death among patients with CKD [6–9]. Studies have shown that sdLDL-C is significantly higher than LDL-C in patients with coronary artery disease and have associated it with the incidence of CVs independently of LDL-C [10, 11]. Accordingly, sdLDL-C seems to be a major and independent CVD risk factor.

To date, there has been no prospective study to assess the association of sdLDL-C with CV onset in patients with CKD, though doing so is newly possible by virtue of innovative, fully automated, homogenous assay techniques that now allow for the routine inspection of a large number of samples. The present study was conducted in effort to determine whether sdLDL-C is an effective (and readily assessed) predictive factor of CVD in CKD patients.

## Methods

### Subjects and study design

A total of 248 non-dialysis CKD patients (Stage 3 to Stage 4) who had no history of treatment with lipid-lowering drugs were enrolled in this study from the Renal Unit of Wujiang Affiliated Hospital of Nantong University between December, 2010 and December, 2012. CKD was diagnosed according to the National Kidney Foundation K/DOQI Guidelines [12]. Exclusion criteria included severe hepatic disease (*n* = 3), infectious disease (*n* = 6), current treatment for malignancy (*n* = 4), and known thyroid disorders (*n* = 2), lost during follow-up (*n* = 13), missing blood examination data (*n* = 3), withdrew consent (*n* = 1), and treatment with lipid-lowering drugsduring the follow-up period (*n* = 71). After these patients were excluded, data from 145 participants was ultimately included in the study. The mean age of participants was 65.2 ± 11.3 years; the individuals ranged in age from 50 to 72 years. Thirty-four of these participants had a verifiable medical history of CVD at the time of enrollment. Of these 34, 3 had a history of stroke, 26 had a history of previous myocardial infarction, and 5 had a history of peripheral vascular disease. Eighty healthy subjects with similar gender, age and sdLDL-C characteristics as the patient groups served as a control group (mean age 64.3 ± 10.8 years, ranging from 52 to 71 years.)

Blood samples were collected and centrifuged immediately after the subjects had fasted for 12 h. The body mass index (BMI), smoking history, and medical history of each participant were collected. A diagnosis of hypertension was given to participants who showed a systolic blood pressure (SBP) ≥140 mmHg, a diastolic blood pressure (DBP) ≥90 mmHg after the first three measurements, or if they had a history of taking anti-hypertensive medications [13]. Diabetes was the diagnosis for a fasting serum glucose registering ≥7.0 mmol/L (126 mg/dL) or for those with a history of anti-diabetes medications [14]. Dyslipidemia was defined as triglyceride (TG) ≥150 (mg/dl), LDL-C ≥140 (mg/dl), or high-density lipoprotein cholesterol (HDL-C) <40 (mg/dl) [15]. We calculated the estimated glomerular filtration rate (eGFR) by using the Modification of Diet in Renal Disease (MDRD) equation: 186 × (serum creatinine)^− 1.154^ × age^− 0.203^ × (0.742 forfemales) [16]. All relevant data was evaluated between October and December of 2014. The endpoints of the present study were date of the first CV onset during the follow-up period, death, or the patient’s last visit to the Wujiang Affiliated Hospital of Nantong University. CVs were defined and registered as listed in Table 1 during the follow-up.

**Table 1:** Cardiovascular events were defined and registered during the follow-up

Cardiovascular events defined	Registered			
	Patients (*n* = 145)		Control (*n* = 80)	
	Non-fatal	Fatal	Non-fatal	Fatal
Heart failure	1	7	0	1
Ischemic stroke	2	0	0	0
Hemorrhagic stroke	4	0	0	0
Acute coronary syndrome	21	1	2	0
Coronary or any peripheral arterial revascularization	18	0	4	0
Total	54		7	
Events%	37.2 %		8.8 %	
*P* ^a^	<0.001			

Data are presented as the number and its percentage (%). Percentage = the number of each individual category divided by n

^a^indicates the comparison of percentage between Patients group and Control group

### Laboratory measurements

We measured the above parameters for all subjects at the onset of CVs yearly during the follow-up period. Laboratory examination results including serum TG, total cholesterol (TC), HDL-C, LDL-C, fasting blood glucose (FBG), apolipoproteins A1 (ApoA1), B (ApoB), and glycated hemoglobin (HbA1c) were measured via HPLC method [17] and hsCRP was estimated via immunoturbidimetry method using a commercial Beckman Synchron DxC 600 fully automated analyser kit (USA). An sdLDL-EX Seiken kit was used for quantitative determination of sdLDL-C in samples according to the manufacturer’s instructions [18].

### Statistical analyses

All statistical analyses were performed in SAS 9.1 software (SAS Institute, Cary, NC, USA). Baseline characteristics were compared between the Event and Non-event groups using a t-test for parametric variables, Wilcoxon tests for non-parametric variables, and Chi-square tests for categorical variables. Differences in cumulative incidence were assessed by log-rank tests in subjects divided into two groups based on the median levels (38 mg/dl) of sdLDL-C. The Cox proportional hazard regression model was used to identify the most significant factors, adjust them by sex, and determine which differed statistically between event and event-free subjects; *P* < 0.05 was considered statistically significant.

## Results

**Fig. 1:**
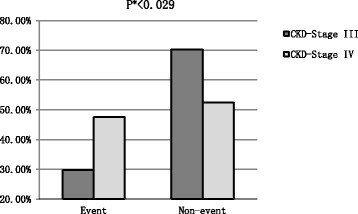
CVD events outcome in CKD-Stage IIIand CKD-StageIV. * indicates that Chi-square analysis was used to compare the distribution of CKD-Stage III and CKD-Stage IV between Event group and Non-event group

As discussed above, Chi-square analysis was used in this study to compare the distribution of CKD-Stage III and CKD-Stage IV between Event and Non-event groups. As shown in Fig. 1, the distribution was notably different; the percentage of CKD-Stage III in the Non-event group was significantly higher than that of the Event group (*p* < 0.029). Table 1 shows that the prevalence rates of CV outcomes were significantly higher in the patient groups than the healthy control group (37.2 % vs. 8.7 %, *P* < 0.001). By comparison of the outcomes of the general and laboratory characteristics between the two groups (Event vs. Non-event) showed that CKD patients with CVs had significantly higher prevalence of diabetes mellitus and hypertension. These differences grew more intense with age, as well. HbA1c, sdLDL-C, LDL-C/HDL-C, and sdLDL-C/LDL-C were higher in the Event group than the Non-event group at baseline. ApoA-I, eGFR, and HDL-C levels were significantly lower in the Event group, and ApoB, Non-HDL-C, and LDL-C were similar between the two groups (Tables 2 and 3).

**Table 2:** The clinical characteristics at baseline of the enrolled patients

Variable	All	CVs	Non- CVs	*P* ^a^
	(*n* = 145)	(*n* = 54)	(*n* = 91)	
Men	113 (77.9 %)	45 (83.3 %)	68 (74.7 %)	0.227
Age(years)	65.2 ± 11.3	68.5 ± 10.5	63.2 ± 11.5	0.006
Body mass index(kg/m^2^)	23.9 ± 3.2	24.5 ± 3.3	23.6 ± 3.2	0.108
History of cardiovascular disease				
Previous myocardial infarction	26 (17.9 %)	10 (18.5 %)	16 (17.6 %)	0.887
Stroke	3 (2.1 %)	1 (1.9 %)	2 (2.2 %)	0.998
Peripheral vascular disease	5 (3.4 %)	2 (3.7 %)	3 (3.3 %)	0.998
Cardiovascular disease risk factors				
Hypertension	116 (80.0 %)	48 (88.9 %)	68 (74.7 %)	0.039
Diabetes mellitus	65 (44.8 %)	32 (59.3 %)	33 (36.3 %)	0.007
Dyslipidemia	123 (84.8 %)	46 (85.2 %)	77 (84.6 %)	0.926
Smoking, current or former	63 (43.4 %)	23 (42.6 %)	40 (44.0 %)	0.873
Family history	26 (17.9 %)	12 (22.2 %)	14 (15.4 %)	0.299
Etiology of CKD				
Diabetes	40 (27.6 %)	14 (25.9 %)	26 (28.6 %)	
Glomerulonephritis	22 (15.2 %)	7 (13.0 %)	15 (16.5 %)	
Hypertension	36 (24.8 %)	16 (29.6 %)	20 (22.0 %)	0.875
Polycystic kidney disease	5 (3.4 %)	2 (3.7 %)	3 (3.3 %)	
Unknown	42 (29.0 %)	15 (27.8 %)	27 (29.7 %)	
Medication				
Angiotensin receptor blocker	65 (44.8 %)	24 (44.4 %)	41 (45.1 %)	0.943
Angiotensin converting enzyme inhibitors	27 (18.6 %)	14 (25.9 %)	13 (14.4 %)	0.082
Insulin	7 (4.8 %)	3 (5.6 %)	4 (4.4 %)	0.753
Antiplatelet	71 (48.9 %)	25 (46.3 %)	46 (50.6 %)	0.620

Data are presented as mean ± SD or the number and its percentage (%). Percentage = the number of each individual category divided by n

*Abbreviation*: *CVs* cardiovascular events

^a^indicates the comparison of mean or percentage between Event group and Non-event group

**Table 3:** The laboratory characteristics at baseline of the enrolled patients

Variable	All	CVs	Non- CVs	*P*
	(*n* = 145)	(*n* = 54)	(*n* = 91)	
LDL–C(mg/dl)	119.2 ± 31.2	120.1 ± 30.2	118.6 ± 32.4	0.783
sdLDL–C(mg/dl)	37.9 ± 21.3	43.4 ± 26.5	34.7 ± 20.2	0.027
lbLDL–C(mg/dl)	80.4 ± 25.5	75.2 ± 25.4	83.5 ± 25.2	0.058
sdLDL–C/LDL–C	0.32 ± 0.13	0.36 ± 0.16	0.29 ± 0.12	0.003
TG(mg/dl)	134.5 (91.3–180.5)	132 (87.5–176.3)	135.3 (93.2–185.6)	0.982
TC(mg/dl)	190.1 ± 34.9	183.9 ± 30.4	193.8 ± 34.8	0.085
HDL–C(mg/dl)	45.7 ± 14.2	42.3 ± 14.8	47.7 ± 13.2	0.024
Non-HDL–C(mg/dl)	142.6 ± 33.6	142.8 ± 31.8	142.5 ± 35.4	0.959
LDL–C/HDL–C	2.84 ± 1.12	3.11 ± 1.16	2.68 ± 1.06	0.024
Apo A-I (mg/dl)	119.0 ± 23.2	111.7 ± 22.7	123.3 ± 22.7	0.003
Apo B(mg/dl)	93.0 ± 22.3	95.4 ± 37.3	91.6 ± 20.7	0.431
FBG (mg/dl)	116.7 ± 36.2	121.6 ± 37.3	113.8 ± 35.8	0.214
HbA1c(%)	6.41 ± 1.23	6.85 ± 1.36	6.15 ± 1.08	<0.001
hs-CRP (mg/dl)	0.39 (0.16–1.52)	0.53 (0.43–1.65)	0.31 (0.15–1.48)	0.069
eGFR(ml/min/1.73 m2)	50.3 ± 16.5	45.6 ± 15.8	53.1 ± 17.7	0.011

Normal Data are presented as mean ± SD, and Median (Q1-Q3) are used for abnormal data

lbLDL-C (mg/dl) = LDL-C (mg/dl) − SdLDL-C (mg/dl)

*Abbreviation*: *LDL-C* low-density lipoprotein cholesterol, *sdLDL-C* small dense LDL-C, *lbLDL-C* large buoyant LDL-C, *TG* triglyceride, *TC* total cholesterol, *HDL-C* high-density lipoprotein cholesterol, *Apo* apolipoprotein, *FBG* fasting blood glucose, *HbAlc* hemoglobin A1c, *eGFR* estimated glomerular filtration rate, *hs-CRP* high sensitivity C-reaction protein, *CVs* cardiovascular events

The unadjusted Cox regression analysis showed that the association was stronger for sdLDL-C/LDL-C than sdLDL-C, age, LDL-C/HDL-C, eGFR, HbA1c, or ApoA-I as they affected the incidence of CVs at the final observation. Increase in LDL-C was not significant in terms of increased risk of CVs, however. After risk adjustment in the multivariate Cox regression analysis, which included the significant predictors above and the marginally significant predictor of CVs in the univariate model, we confirmed that sdLDL-C/LDL-C had stronger impact than sdLDL-C, age, or HbA1c at final observation. Moreover, LDL-C/HDL-C was not found to be a statistically significant independent risk factor for CVs (Table 4).

**Table 4:** Univariate and multivariate Cox,s proportional hazard analysis predicting for cardiovascular events

Variable	Univariate model	Multivariate model	
	HR 95 % CI	Model 1	Model 2
		HR 95 % CI	HR 95 % CI
Age	**1.251 (1.145**–**1.590)**	**1.204 (1.032**–**1.578)**	**1.214 (1.143**–**1.580)**
Men	0.801 (0.488–1.313)	0.774 (0.555–1.086)	0.776 (0.553–1.087)
sdLDL–C	**1.294 (1.026**–**1.633)**	**1.275 (1.016**–**1.596)**	**1.280 (1.020**–**1.624)**
sdLDL–C/LDL–C	**2.526 (1.653**–**22.5904)**	**2.450 (1.420**–**15.630)**	**2.445 (1.402**–**14.521)**
LDL–C	1.015 (0.908–1.131)	-	-
HDL–C	0.721 (0.450–1.130)	0.822 (0.475–1.346)	-
Non-HDL–C	1.045 (0.912–1.118)	-	-
LDL–C/HDL–C	**1.167 (1.013**–**1.323)**	1.012 (0.913–1.124)	-
Apo A-I	**0.963 (0.941**–**0.992)**	-	0.982 (0.943–1.142)
HbA1c	**1.215 (1.133**–**1.684)**	**1.204 (1.101**–**1.504)**	**1.135 (1.103**–**1.497)**
eGFR	**0.965 (0.926**–**0.992)**	0.979 (0.936–1.002)	-
hs-CRP	1.007 (0.912–1.113)	1.071 (0.941–1.085)	-

Abbreviations are the same as those for Table 3. Model1 was adjusted age, gender, sdLDL–C, sdLDL–C/LDL–C,HDL–C, LDL–C/HDL–C, HbA1c, eGFR, hs-CRP. Model 2 was adjusted age, gender, sdLDL–C, ApoA-I,sdLDL–C/LDL–C,HbA1c. Bold numbers: statistically significant

**Fig. 2:**
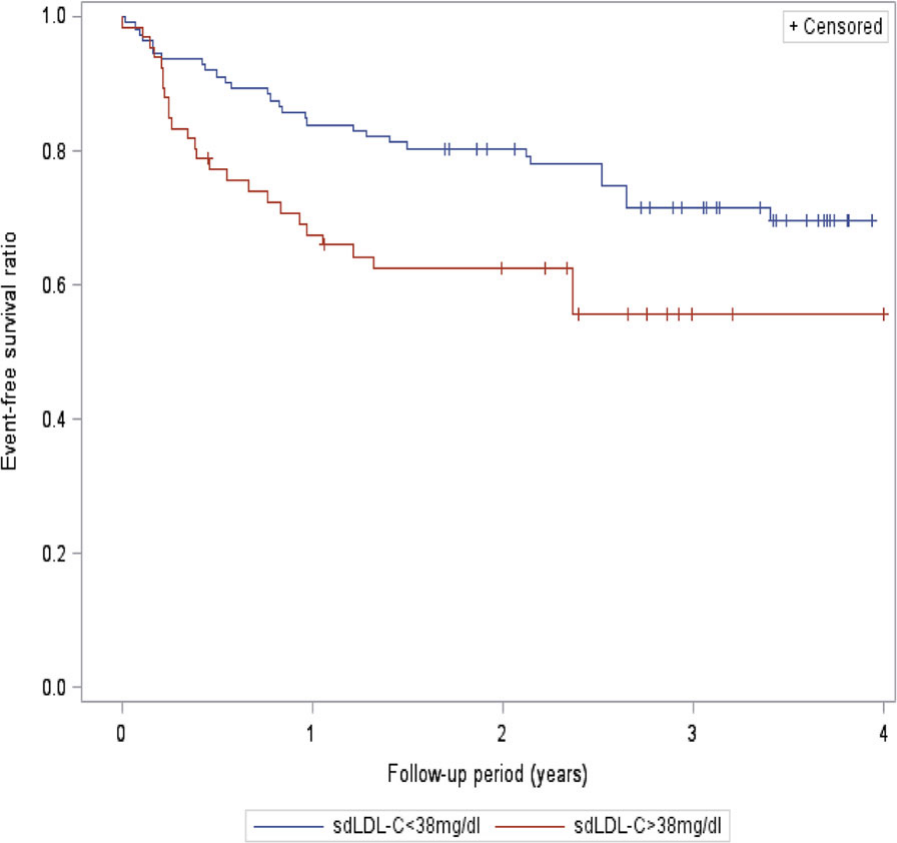
Kaplan-Meier event-free survival curves according to sdLDL-C levels were above or below the median (38 mg/dl)

**Fig. 3:**
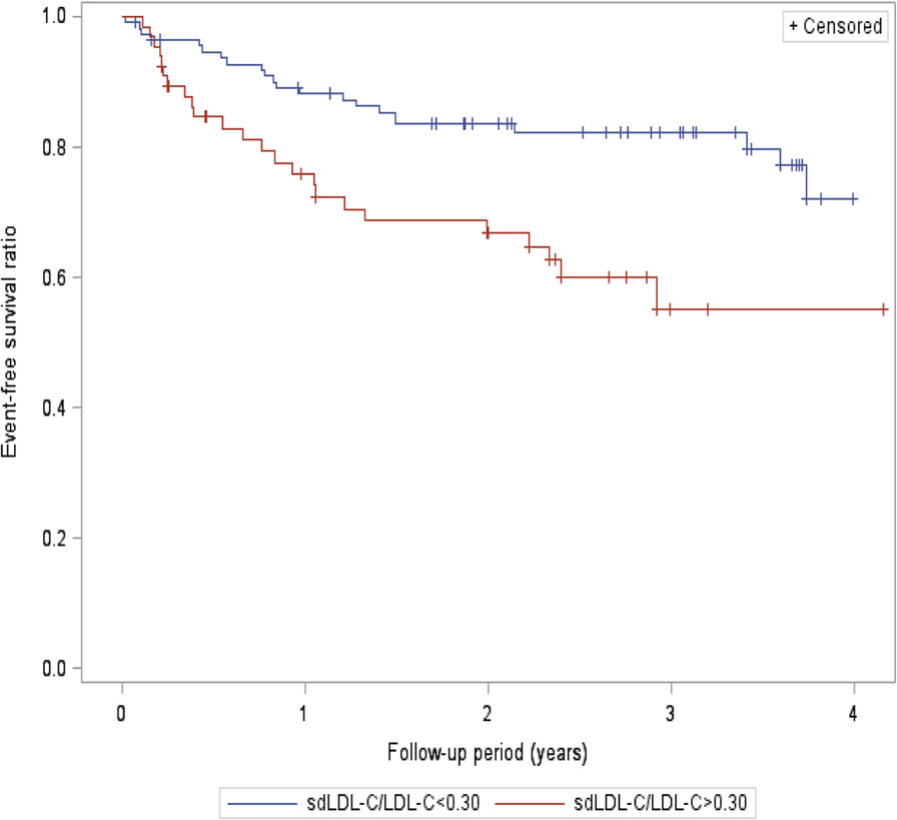
Kaplan-Meier event-free survival curves according to sdLDL-C/LDL-C ratio were above or below the median (0.3)

During a median follow-up period of 2.3 years, Kaplan-Meier survival analysis results showed that sdLDL-C levels were significantly associated with CV incidence in our participants: Patients with sdLDL-C >38 mg/dl levels were at increased risk for CVs (logrank: 4.375, *P* = 0.037) as shown in Fig. 2, as were patients with sdLDL-C/LDL-C ratio >0.3 (logrank: 11.94, *P* = 0.018) as shown in Fig. 3.

## Discussion

The results of multivariate Cox regression analysis, which included the significant predictors and the marginally significant predictors of CVs in the univariate model, showed that elevated sdLDL-C concentration and sdLDL-C/LDL-C can be associated with increased CVD risk in CKD patients. We also found that HR was significant after multivariable adjustment and by analysis including gender, age, HbA1c, eGFR, hs-CRP, and other lipid risk factors in Models 1 and 2; sdLDL-C, HbA1c, and sdLDL-C/LDL-C concentrations did remain an independent risk predictor for CVs. In our study, participants with elevated HbA1c levels were the most at risk for CVs, suggesting that without proper diabetes control, there is an increased risk for CVs. An increase in sdLDL-C/LDL-C also indicated CV risk. An imbalance of cholesterol-poor sdLDL and cholesterol-rich large LDL led to a risk of CVs for patients who did not receive any lipid-lowering medications during the follow-up period. There is evidence that sdLDL formation is related to postprandial hyperglycemia and hyperlipidemia, both of which can lead to CVs. Additionally, the sdLDL-C/LDL-C ratio is another risk factor for CVs in CKD patients. Patients with CKD have elevated sdLDL-C/LDL-C, over sdLDL-C, and declining eGFR, are at risk for CVs.

Patients with CKD have significantly increased risk for cardiovascular complications. Traditional risk factors fail to fully explain the high incidence of CVD in CKD patients. Similarly, traditional lipid measures are not sufficient for predicting cardiovascular outcomes in CKD patients [1, 19–21]. In accordance with the results presented here, previous studies have shown that sdLDL-C is significantly higher than LDL-C in patients with coronary artery disease and have associated it with the incidence of CVs independently of LDL-C [10, 11]. As discussed above, we also failed to observe any significant impact on CV risk with increased LDL-C. Our multiple regression analysis results also suggested that the common blood lipid and lipoprotein index measures fail to accurately predict CVs, and that increased sdLDL is apparently the most effective atherogenic risk factor in CKD patients [22–25].

It is worth mentioning that sdLDL, which has greater susceptibility to oxidation, is already regarded as a risk marker for CVD [26–32]. There is scientific evidence that sdLDL particles are highly atherogenic and can be a biomarker of CVD [30–32]. Traditional methods of detecting sdLDL are generally ineffective, however, as they require laborious and lengthy assay processes [33, 34]. The results of this study support the role of sdLDL-C in regards to CVD risk, and also suggest that routine detection is indeed possible via a new, fully automated, homogenous assay technique.

This study had several potential limitations. First, patients with CVs had more diabetes and hypertension, and these factors were not evaluated. Second, the effects of insulin therapy were not investigated. The small sample size and relatively brief follow-up period were also less than ideal. Finally, the cohort may not be completely representative of the CKD population due to the exclusion of patients treated with lipid-lowering medication. Future studies should focus on resolving these issues, especially with a larger sample size.

## Conclusion

The results of this study indicated that for patients suffering CKD, a significant relationship exists between an elevated sdLDL-C concentration and the risk of cardiovascular disease.

## Abbreviations

Apo: Apolipoprotein
CKD: Chronic kidney disease
CVD: Cardiovascular disease
eGFR: Estimated glomerular filtration rate
FBG: Fasting blood glucose
HbAlc: Hemoglobin A1c
HDL: High-density lipoprotein
HDL-C: HDL cholesterol
HPLC: High performance liquid chromatography
hs-CRP: High sensitivity C-reaction protein
lbLDL: Large buoyant LDL
LDL: Low-density lipoprotein
LDL-C: LDL cholesterol
sdLDL: Small dense LDL
sdLDL-C: Small dense low-density lipoprotein cholesterol
TC: Total cholesterol
TG: Triglyceride
